# Circulating cell-free DNA methylation mirrors alterations in cerebral patterns in epilepsy

**DOI:** 10.1186/s13148-022-01416-2

**Published:** 2022-12-28

**Authors:** Ricardo Martins-Ferreira, Bárbara Leal, João Chaves, Laura Ciudad, Raquel Samões, António Martins da Silva, Paulo Pinho Costa, Esteban Ballestar

**Affiliations:** 1 Epigenetics and Immune Disease Group, Josep Carreras Research Institute (IJC), 08916 Badalona, Barcelona Spain; 2 Immunogenetics Laboratory, Molecular Pathology and Immunology Instituto de Ciências Biomédicas Abel Salazar – Universidade do Porto (ICBAS-UPorto), Rua Jorge Viterbo Ferreira, 228, 4050-313 Porto, Portugal; 3 Autoimmunity and Neuroscience Group, Unit for Multidisciplinary Research in Biomedicine (UMIB), ICBAS-UPorto, Rua Jorge Viterbo Ferreira, 228, 4050-313 Porto, Portugal; 4 Laboratório Para a Investigação Integrativa e Translacional em Saúde Populacional (ITR), Porto, Portugal; 5 Neurology Service, Hospital de Santo António - Centro Hospitalar Universitário do Porto (HSA-CHUP), Porto, Portugal; 6 Neurophysiology Service, HSA-CHUP, Porto, Portugal; 7 Department of Human Genetics, Instituto Nacional de Saúde Dr. Ricardo Jorge, Porto, Portugal; 8 Epigenetics in Inflammatory and Metabolic Diseases Laboratory, Health Science Center (HSC), East China Normal University (ECNU), Shanghai, 200241 China

**Keywords:** Cell-free DNA, DNA methylation, Epilepsy, Biomarker

## Abstract

**Background:**

DNA methylation profiling of circulating cell-free DNA (cfDNA) has rapidly become a promising strategy for biomarker identification and development. The cell-type-specific nature of DNA methylation patterns and the direct relationship between cfDNA and apoptosis can potentially be used non-invasively to predict local alterations. In addition, direct detection of altered DNA methylation patterns performs well as a biomarker. In a previous study, we demonstrated marked DNA methylation alterations in brain tissue from patients with mesial temporal lobe epilepsy with hippocampal sclerosis (MTLE–HS).

**Results:**

We performed DNA methylation profiling in cfDNA isolated from the serum of MTLE patients and healthy controls using BeadChip arrays followed by systematic bioinformatic analysis including deconvolution analysis and integration with DNase accessibility data sets. Differential cfDNA methylation analysis showed an overrepresentation of gene ontology terms and transcription factors related to central nervous system function and regulation. Deconvolution analysis of the DNA methylation data sets ruled out the possibility that the observed differences were due to changes in the proportional contribution of cortical neurons in cfDNA. Moreover, we found no overrepresentation of neuron- or glia-specific patterns in the described cfDNA methylation patterns. However, the MTLE–HS cfDNA methylation patterns featured a significant overrepresentation of the epileptic DNA methylation alterations previously observed in the hippocampus.

**Conclusions:**

Our results support the use of cfDNA methylation profiling as a rational approach to seeking non-invasive and reproducible epilepsy biomarkers.

**Supplementary Information:**

The online version contains supplementary material available at 10.1186/s13148-022-01416-2.

## Background

Cell-free DNA (cfDNA) consists of small DNA fragments released into the peripheral blood, predominantly as a result of apoptosis [1]. This is substantiated by the consistent correspondence between the length of human circulating cfDNA (167 bp) and the length of DNA wrapped around a nucleosome (~ 147 bp) plus linker regions, which suggest the action of endonucleases. Apoptotic DNA degradation is mediated by caspase-activated DNase (CAD), which lacks exonuclease activity, and therefore can only cleave DNA in inter-nucleosomal regions [1, 2]. The evaluation of DNA methylation of cfDNA has been used to estimate tissue or cell of origin, and to non-invasively track ongoing cell death occurring anywhere in the body, based on the cell-specific nature of DNA methylation [3]. The use of this approach is spreading in cancer studies [4–8]. Direct detection of altered DNA methylation patterns under pathological conditions, regardless of cell or tissue contribution, has been thoroughly examined to determine its value as a strategy for searching for biomarkers [9–14]

The identification and development of epilepsy and epileptogenesis biomarkers are of inherent interest [15], but progress has been slower than in other settings, including other neurodegenerative pathologies like Alzheimer’s disease (AD). This can be attributed to the low accessibility to pathological tissue and the complexity and variability within the spectrum of epilepsy syndromes. Nevertheless, nucleic acid-based biomarkers, predominantly microRNAs, are promising [16].

Here, we obtained the DNA methylation profiles of serum cfDNA samples from mesial temporal lobe epilepsy (MTLE) patients and compared them with those obtained from healthy controls. MTLE is commonly associated with severe neuronal cell death, termed hippocampal sclerosis (HS) [17]. We hypothesized that the analysis of the cfDNA methylome in MTLE–HS patients could serve as a predictive, diagnostic or prognostic tool of neuronal cell death estimation. Furthermore, a direct comparison of the DNA methylation profile between patients and healthy controls could potentially fill the gap in peripheral biomarker development in epilepsy. Our results were not able to identify a significant increase in the proportion of cfDNA derived from brain. However, the cfDNA methylomes reflect a significant enrichment of epileptic patterns overlapping with those described in the hippocampus of MTLE patients.

## Results

### Estimation of cell of origin proportions in serum of MTLE and controls based on cfDNA methylation

**Fig. 1:**
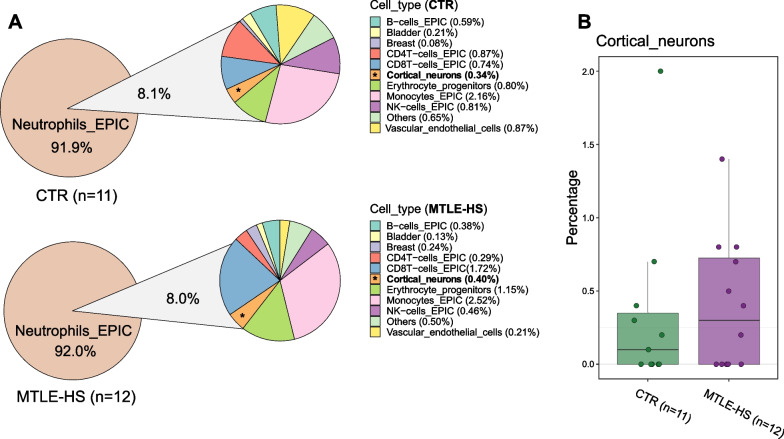
Estimated percentage contribution of each cell and tissue type from the meth_atlas deconvolution tool for the 11 control (CTR) and 12 MTLE serum samples analysed. **A** Pie chart of the mean estimated percentage contribution for each group. Percentage of cfDNA originating in cortical neurons is highlighted in bold and with an asterisk. **B** Boxplot representation of the individual estimated proportion of cfDNA originated by cortical neurons within each study group (*p* = 0.54, Wilcoxon test)

First, we generated the DNA methylation profiles of serum cfDNA samples of MTLE and healthy controls (Table 1 and Additional file 4: Table S1) using BeadChip arrays. To estimate the cell of origin, we used the deconvolution algorithm designed for this purpose by Moss et al. [8], in relation to a 7890-CpG reference matrix, accounting for 25 tissue and cell types (Fig. 1A, Additional file 1: Figure S1 and Additional file 5: Table S2). In agreement with other studies, including the original source of the algorithm, hematopoietic cells were the main contributors. We also noted contributions from non-blood cell or tissue types, including bladder, breast, vascular endothelial cells and cortical neurons. Significant differences in the cell-of-origin proportion between controls and MTLE patients were only observed for vascular endothelial cells (*p* = 0.02, Wilcoxon test) (Additional file 1: Figure S1B). No significant differences in the cortical neuronal origin were observed between patients and controls (*p* = 0.54, Wilcoxon) (Fig. 1B). We noted a striking predominance of neutrophil contribution in both groups, with a mean percentage contribution of approximately 92% across all samples (Fig. 1A and Additional file 1: Figure S1); this is a higher value than that reported by Moss and colleagues. This might be due to the use of serum samples, instead of plasma samples, as they used. We can speculate that the predominance of neutrophil contribution is associated with increased coagulation-related NETosis during sample collection.

**Table 1:** Clinicodemographic characterization of the studied populations

	Controls	MTLE	*p*
*n*	11	12	–
% Female (n)	81.8 (9)	75.0 (9)	1.000
Age, years (mean ± SD)	38.9 ± 8.4	44.8 ± 11.4	0.2063
Age of onset, years (mean ± SD)	–	14.0 ± 13.6	–
Epilepsy duration (mean ± SD)	–	30.8 ± 14.5	–
% of pharmacoresistant (n)	–	83.3 (10)	–
% of FS history	–	75.0 (9)	–

*MTLE* Mesial temporal lobe epilepsy, *FS* Febrile seizures, *SD* Standard deviation

### Differentially methylated regions (DMRs) in cfDNA

**Fig. 2:**
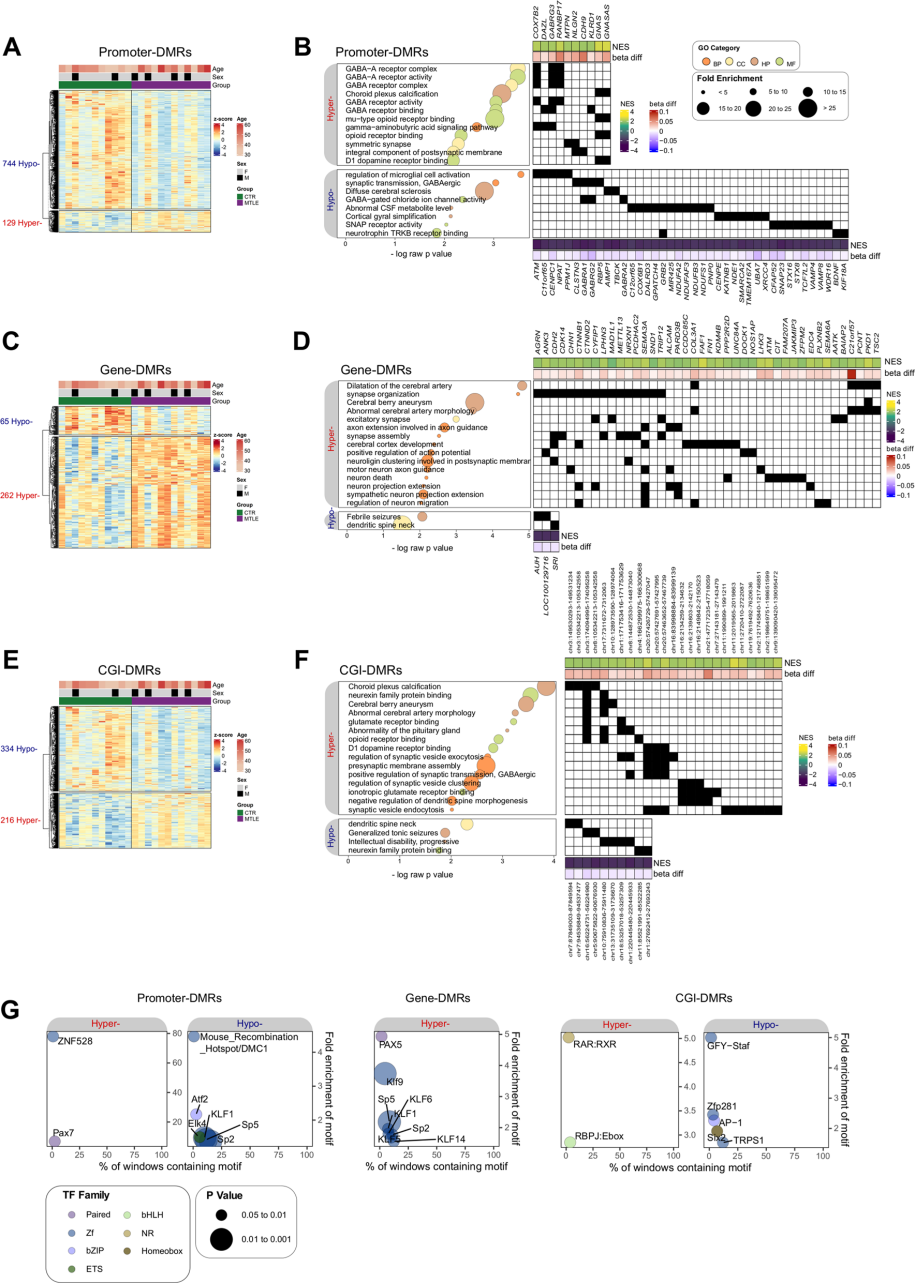
Heatmap representation of DNA methylation of promoter (**A**), gene (**C**), and CGI-DMRs (**E**) in 12 MTLE patients compared with 11 controls. The DNA methylation value of each DMR corresponds to the mean beta value across all single-nucleotide positions encompassed by the DMR. Each individual is annotated with respect to age and sex. GO enrichment analysis of hypermethylated and hypomethylated DMRs across the promoter (**B**), gene (**D**), and CGI-DMR (**F**) types, showing the most biologically relevant terms. GO categories include biological process (BP), molecular function (MF), cellular component (CC) and human phenotype (HP). Enrichment is represented by *p* value and fold enrichment. For each GO term, the corresponding DMR hits were identified in a heatmap. NES and beta difference values (MTLE-CTR) for each gene were included to represent the degree of differential methylation. **G** HOMER binding motif enrichment of hypomethylated and hypermethylated DMRs. Colour depicts the transcription factor family; bubble size indicates the level of significance (*p*)

We then determined DMRs, using the mCSEA algorithm, between MTLE and healthy control cfDNA. Three sets of DMRs, located in promoters, gene bodies and CpG islands (CGIs), were calculated. We identified 873 significant promoter DMRs (744 hypomethylated and 129 hypermethylated in MTLE relative to controls) (Fig. 2A and Additional file 6: Table S3). Gene ontology (GO) analysis of the hypermethylated and hypomethylated DMR clusters demonstrated enrichment of CNS-related terms, including some associated with GABAergic pathways, synaptic transmission, microglia activation and neurotrophin receptor binding. A thorough inspection of the DMRs associated with these GO categories included biologically relevant promoters such as those associated with the *GABRG3* and *CDH9* genes (hypermethylated), and those at the *GABRA1, GABRA2, GABRG2* and *BDNF* genes (hypomethylated) (Fig. 2B). GABAergic receptors represent key constituents of the CNS. γ-aminobutyric acid (GABA) is the main inhibitory neurotransmitter in the cerebral cortex and disruption in the excitatory/inhibitory balance has long been associated with seizure development [18]. On this basis, genetic variability related to GABAergic subunits has shown potentially causal epileptogenic effects [19]. *BDNF* encodes the brain-derived neurotrophic factor, one of the most prominent members of the neurotrophin family, which has a wide range of functions, encompassing regulation of neuronal development and synaptic plasticity [20]. The regulation of the *BDNF* gene in neurons has long been associated with DNA methylation-related mechanisms [21]. It has been described as being overexpressed in epilepsy [22–26]. Moreover, the DNA methylation status of its promoter regions has been explored [27–29], revealing a marked tendency towards demethylation.

We also described 327 gene-body DMRs (65 hypomethylated and 262 hypermethylated in MTLE compared with healthy controls) (Fig. 2C and Additional file 6: Table S3). Multiple CNS-related GO categories were also found to be associated with obtained DMRs. These included terms associated with synaptic assembly and organization (hypermethylated) and febrile seizures (hypomethylated) (Fig. 2D).

We found 550 significant CGI-DMRs (334 hypomethylated and 216 hypermethylated) (Fig. 2E and Additional file 6: Table S3). GO analysis was consistent with that described above, with the enrichment of multiple mechanisms of potential relevance in neuropathology (Fig. 2F).

We also examined the TF binding motif enrichment of the generated DMRs (Fig. 2G). To analyse the potential CNS-specific activity of the enriched TFs, we used data from the HPA repository. Many of the enriched TFs were consistently expressed across different brain regions (Additional file 2: Figure S2A). Moreover, we searched for associations of the genes coding the enriched TFs with HPA tissue clusters and HPA single-cell clusters. The most prominent results were the high confidence levels of *ZNF528* and *RARB* in *Cluster 73—Brain: Transcription regulation* (RNA HPA tissue expression cluster) (Additional file 2: Figure S2B) and *Cluster 8—Neurons & Oligodendrocytes: Synaptic function* (RNA HPA single-cell expression cluster) (Additional file 2: Figure S2C), respectively. This suggests possible brain specificity in the establishment of these DNA methylation patterns in cfDNA of MTLE patients.

### cfDNA DMRs in MTLE show enrichment of brain epileptic DMRs but not of neuron- or glia-specific methylation patterns

**Fig. 3:**
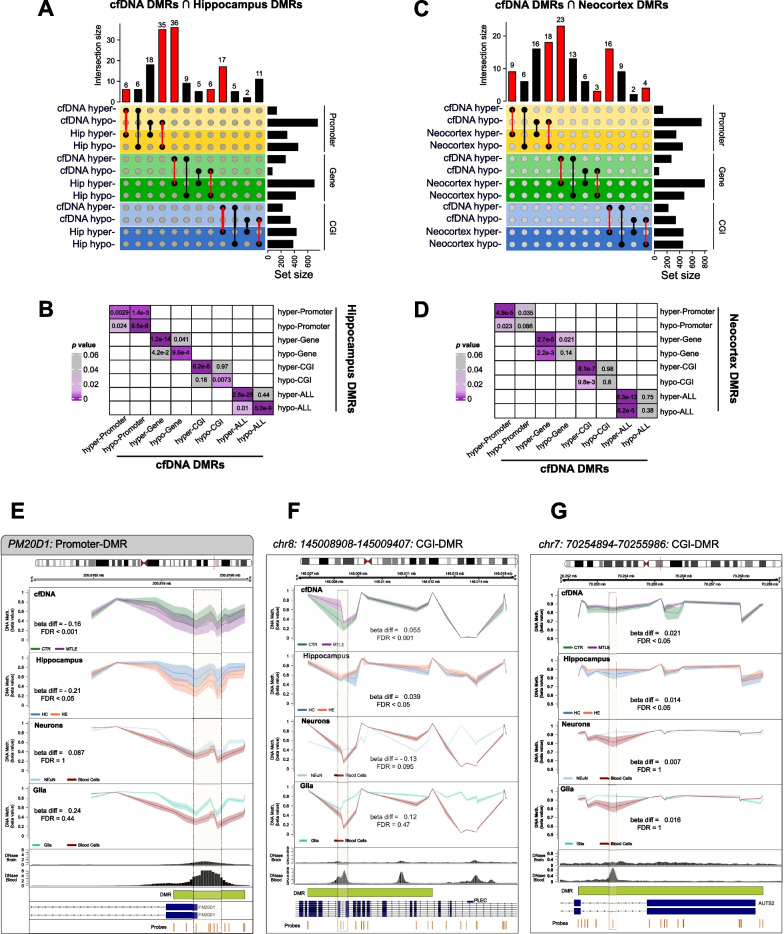
**A** Representation of the overlap between cfDNA DMRs in MTLE and hippocampal DMRs in MTLE. The sets of DMRs overlapped separately in relation to the type of DMR (promoter, gene, CGI) and the methylation behaviour (hypermethylated and hypomethylated). Red bars and lines indicate the overlap of DMRs with coincidence in the direction of change. **B** Heatmap matrix representation of the *p* values associated with the Fisher’s exact test of the overlaps between DMRs in cfDNA and in the hippocampus. **C** Representation of the overlap between cfDNA DMRs in MTLE and neocortical DMRs in MTLE. The sets of DMRs overlapped separately in relation to the type of DMR (promoter, gene, CGI) and the methylation behaviour (hypermethylated and hypomethylated). Red bars and lines indicate the overlap of DMRs with coincidence in the direction of change. **D** Heatmap matrix representation of the *p* values associated with the Fisher’s exact test of the overlaps between DMRs in cfDNA and in the neocortex. **E**–**G** Graphical representation of the DNA methylation of the individual probes in cfDNA, hippocampus, neurons *vs*. blood cells, and glia *vs.* blood cells, and DNase-seq hypersensitivity in brain tissue and blood cells, corresponding to the DMRs located at the promoter of the *PM20D1* gene (**E**), at the chr8:145,008,908–145,009,407 CGI (**F**) and at the chr7: 70,254,894–70,255,986 CGI (**G**). DNA methylation is presented as beta values. Beta diff is the mean difference of the beta values of all individual probes in the DMR for each comparison. FDR corresponds to the Bonferroni-adjusted *p* value emerging from the *mCSEA* DMR calculation. The genomic location of each DMR is highlighted by a red line in the respective chromosome. The DMRs (green) and the individual probes (orange) are presented in relation to the annotated genes in the UCSC Ref Seq

Previously, our group described significantly altered DNA methylation patterns in the hippocampus and neocortex of MTLE–HS patients compared with autopsied controls without neuropathology [30]. These include 2650 DMRs in the hippocampus (736 promoter, 1111 gene and 803 CGI) (Additional file 3: Figure S3A) and 2950 DMRs in the adjacent neocortex (785 promoter, 1264 gene and 901 CGI) (Additional file 3: Figure S3B). The overlap of cfDNA DMRs with hippocampal DMRs in MTLE patients was significant (Fig. 3A, B). The coincidence of the direction of methylation change (hypermethylated or hypomethylated in both cfDNA and hippocampus) reinforced the relevance of the overlap of DMRs. Regarding the comparison between cfDNA and neocortex DMRs, there was a marked enrichment between hypermethylated DMRs, but we found no significant overlap between hypomethylated DMRs (Fig. 3C, D).

We considered whether the dimensionality reduction achieved by genome-wide deconvolution algorithms was too broad and overlooked specific DNA methylation changes that could indicate differences in the cell-specific contribution. Cell of origin may be represented by a small subset of target regions. Therefore, we also attempted to determine whether the DMRs obtained in cfDNA of MTLE patients are enriched in neuron- or glia-specific DNA methylation patterns. We used public EPIC data consisting of neuron and glia samples and blood cells (monocytes, neutrophils, B cells, CD4+ T cells, CD8+ T cells and NK cells). We identified 5943 neuron-specific DMRs (1504 promoter, 2174 gene, 2265 CGI) (Additional file 3: Figure S3C) and 3523 glia-specific DMRs (1038 promoter, 1444 gene, 1041 CGI) (Additional file 3: Figure S3D). We did not find any significant overlap between cfDNA DMRs from the MTLE/CTR comparison or neuron-specific or glia-specific DMRs with concordant behaviour. In fact, greater enrichment was observed for DMRs with opposite behaviour (e.g. hypermethylated promoter cfDNA in MTLE cfDNA and hypomethylated promoter neuron-specific DMRs) (Additional file 3: Figure S3E–H). These results therefore imply that the differences in DNA methylation observed in the cfDNA of patients compared with controls do not appear to be a consequence of increased circulating neuronal or glial DNA.

### DNA accessibility may provide clues about the origin of cfDNA DMRs

At this point, we have demonstrated that DNA methylation alterations in the MTLE brain are, to some extent, replicable in circulating cfDNA. However, we are yet to determine which cells are the source of the fragments carrying such differentially methylated patterns. We next investigated the potential origin of the fragments carrying the identified cfDNA DMRs by using public chromatin accessibility data, namely DNase-seq. It can be assumed that euchromatic regions (open chromatin) would be highly degraded and the resulting fragments would be too small to be detected in circulating cfDNA [31]. We were able to individualize DMRs with higher chromatin accessibility in blood cells than in brain tissue (Fig. 3E–F). We found lower DNA methylation levels in the promoter of the *PM20D1* gene in both cfDNA and hippocampus of MTLE patients. This hypomethylation pattern is neither neuron- nor glia-specific. DNA accessibility, however, is high in blood cells but low in brain tissue (Fig. 3E). We identified two more DMRs, localized in CpG islands located at the *PLEC* and *AUTS2* genes, that show hypermethylation in cfDNA and hippocampus of patients compared with controls. Although the DMR does not show overall significant hypermethylation in neurons or glia, individual CpG probes that overlap with the blood euchromatin region have higher DNA methylation levels in neurons and glia than in blood cells. Both these regions also present higher accessibility in blood cells than in brain tissue (Fig. 3F–G).

## Discussion

Only two previous studies have analysed cfDNA in the context of epilepsy, and they only measured total cfDNA concentration levels [32, 33]. MTLE is the most incident-focal epilepsy in adults, which, together with its high pharmacoresistant rates, makes it one of the most widely studied epilepsy syndromes. DNA methylation has been thoroughly explored within epileptic brain tissue. In a recent study by our group [30], we described major DNA methylation alterations in the hippocampus, which is the focus of the epilepsy and the region of the lesion [17], and in the adjacent neocortical areas of MTLE patients, comparing these with the state in non-epileptic controls. Our present study demonstrates the presence of DNA methylation alterations in the cfDNA of MTLE patients. We have identified a representation of CNS-related genes within the identified alterations. The functional enrichment analysis of the distinct cfDNA methylation patterns observed in MTLE supports its possible origin by highlighting multiple genes, pathways and regulatory transcription factors associated with the CNS paradigm. We found that the epileptic patterns described in hippocampus and neocortex tissue were also significantly enriched in circulating cfDNA. However, a clearer overlap was demonstrated in the hippocampus, which is further evidence of the primary pathological nature of this region. This concurs with the findings of previous studies that cfDNA from serum and plasma can contain tissue-pathological DNA methylation alterations [34–36].

One aspect that remains unresolved is the source of the cfDNA fragments bearing those epileptic patterns. Given that blood cells from epilepsy patients and controls also present altered DNA methylation patterns [37, 38], epileptic patterns observed in cfDNA could originate from blood cells. Furthermore, the DNA methylation profiles of peripheral tissues, including blood, positively correlate with brain tissue in epilepsy patients [39]. It has been proposed that chromatin accessibility states may help to infer the origin of cfDNA [31]. Highly accessible euchromatic regions are likely to be highly degraded, and the resulting fragment would be too small to be detected in circulation. In the three examples of DMR-containing sequences that we have presented, chromatin accessibility is high in blood cells whereas it shows a more compact state in brain tissue. We may assume that the cfDNA fragments carrying those epileptic patterns, coincident in cfDNA and hippocampus, are more likely to originate in brain tissue rather than in blood cells. *PM20D1*, encoding an N-fatty acyl amino acid (NAAs) synthase/hydrolase, is located within a Parkinson’s susceptibility locus [40]. It has been demonstrated that *PM20D1* is both an expression and methylation quantitative trait locus in AD, with a direct influence on molecular and behaviour pathological features [41]. In AD blood samples, a U-shaped model has been proposed in which DNA methylation of this region is decreased in early stages and reverses with progression towards late AD [42]. Plectin (*PLEC*) is a plakin responsible for linking elements of the cytoskeleton. In the CNS, *PLEC* has been shown to be predominantly expressed in pia/glia and endothelia/glia junctions [43], where it is paramount for the structural and functional integrity of the BBB and the pial surface [44]. In TLE, plectin is upregulated in astrocytes located at the sclerotic hippocampus [45]. *AUTS2* is a well-known risk gene for autism spectrum (ASD) but also for other neurodevelopmental disorders, including epilepsy [46]. The autism susceptibility candidate 2 (AUTS2) gene links with PRC1 (polycomb repressive complex 1), a known epigenetic regulator, and together are responsible for the transcription activation of genes associated with neurodevelopment [47].

We did not observe any enhancement of cortical neuron-derived cfDNA in our patients. One must consider the potential lack of precision of the current deconvolution tools in estimating the contribution of brain cells. Moss et al. [8], for instance, used three cortical neuron samples to develop their reference matrix. It is of inherent interest to develop more precise algorithms which would account for, as far as possible, the whole complexity of the CNS spectrum by including multiple cell types (e.g. excitatory and inhibitory neurons, oligodendrocytes, OPCs, astrocytes, microglia) and also take regional variability into account. Additionally, the eventual release of brain-derived cfDNA in MTLE may be an acute event. In fact, Chatterton et al. reported an increase in the release of neuronal and glial cfDNA in the plasma of entry personnel (breachers) during explosive training, on the day that participants were exposed to higher pressures, after which it promptly decreased [31]. In MTLE, such events could occur immediately following the seizure. However, pertinent information such as time elapsed since the last seizure, or the frequency of seizures was not taken into account and remains a limitation to this study.

## Conclusion

Our study shows that the analysis of cfDNA methylation in epilepsy has predictive potential. To follow this up, complementary studies are needed that exploit this emerging field to its full potential. Artificial intelligence and machine learning predictive models, whose performance has been tested in cfDNA [35, 36, 48], would be an important next step in the follow-up of the results presented here. We consider cfDNA methylation to be a promising tool with which to pursue the ultimate goal of reducing patients’ burden through early diagnosis, proper monitoring of the progressive nature of the disease, and a better understanding of the causes of epileptic refractoriness.

## Methods

### Study population

The MTLE patients included in this study were followed at the Reference Epilepsy Research Centre of *Hospital de Santo António—Centro Hospitalar e Universitário do Porto* (HSA-CHUP) (Table 1 and Additional file 4: Table S1). The diagnosis was based on clinical and electrophysiological data (electroencephalogram (EEG) and/or video-EEG monitoring) and brain MRI (minimum 1.5 T), as defined by Wieser [49]. A definition of HS by brain MRI required the detection of atrophy, T2 hyperintensity signal and altered internal structure on one or both hippocampi, associated or not with other imaging criteria such as ipsilateral fornix atrophy, ipsilateral mammillary body atrophy or ipsilateral entorhinal abnormalities. Visual and/or verbal memory impairment were not considered exclusion criteria. However, patients with other neurological abnormalities were not included. At the time of the study, all patients were receiving pharmacological treatment (monotherapy or polytherapy). The control population comprised healthy individuals who were ethnically matched and from the same geographical area, and who had been voluntarily recruited from blood donors. Individuals with any neurological condition or a positive family history were excluded.

### Serum collection and DNA extraction

Peripheral blood was collected in *Vacuette*^®^ tubes without anticoagulant and centrifuged at 490 g for 20 min. Collected serum aliquots were stored at − 20 °C. Only samples processed within 4 h of collection were included. DNA was extracted from approximately 1 mL of serum using the QIAmp^®^ MinElute^®^ ccfDNA Mini Kit (Qiagen), following the manufacturer’s instructions.

### DNA methylation profiling

Extracted genomic DNA was quantified using a Qubit DNA Assay Kit (Cat. No. 10146592) in a Qubit 2.0 Fluorometer (Life Technologies, CA, USA). CfDNA samples from twelve MTLE patients (8F, 4 M; 44.8 ± 11.4 years old) and eleven controls (9F, 2 M; 38.9 ± 8.4 years old) were profiled. All DNA extracted from each sample (44.72–238.95 ng) was bisulphite-converted using the EZ DNA Methylation-Gold™ Kit (Zymo Research, Irvine, CA, USA), following the manufacturer’s instructions. Converted DNA was hybridized in Infinium MethylationEPIC BeadChip arrays (Illumina), following the manufacturer’s instructions. The arrays encompass > 850,000 single-nucleotide methylation sites and cover 99% of the annotated reference sequence (RefSeq) genes. Fluorescence intensities were imaged using a BeadArray Reader (Illumina), and images were processed and intensities measured as previously described [50]. A combination of the Cy3 and Cy5 fluorescence intensities of the methylated and unmethylated alleles was used to obtain each methylation data point. Background intensity was computed from a set of negative controls and subtracted from each data point. Beta values were used to illustrate methylation. Values can range between zero (0% methylation) and one (100% methylation) and represent the ratio of the methylated probe intensity to the overall intensity (sum of the methylated and unmethylated probe intensities). *M* values were calculated as the log_2_ ratio of the intensities of the methylated and unmethylated probes. *M* values were used for statistical purposes, because beta values are heteroskedastic for highly methylated and unmethylated CpGs [51]. Methylation data were analysed in the R statistical environment. The *shinyÉpico* web interface [52], based on *minfi* [53] and *limma* [54] pipelines, was used for array processing, normalization and differential methylation calculation.

### Deconvolution of cell/tissue of origin

The cell or tissue of origin was estimated with the meth_atlas deconvolution algorithm (https://github.com/nloyfer/meth_atlas), which estimates the proportion of origin for a total of 25 tissue and cell types based on 450 k and EPIC data [8]. DNA methylation profiles from serum cfDNA of epileptic patients and controls were processed in accordance with the original study. Normalization was performed with the *preprocessIllumina* function. Probes with a detection significance of *p* > 0.01 were excluded, as were those mapping to sex chromosomes. Deconvolution was performed using the Python-based method in relation to the supplied reference atlas matrix composed of 7890 sites.

### Calculation of cfDNA differentially methylated regions (DMRs)

Methylation data were normalized with the *Noob* + *Quantile* functions to assess the differentials. Probes with a detection *p* < 0.01 were filtered out, as were positions located in the X and Y chromosomes and/or overlapping with SNPs. CpHs were retained, based on evidence that neurons, unlike other CNS cells, present CpH as their dominant DNA methylation mark [55]. A total of 783,351 individual positions were obtained after normalization and filtering. DMRs were calculated using the *mCSEA* (methylated CpGs Set Enrichment Analysis) package [56]. The eBayes results of *limma*, sorted by *t*-statistics, were used as input. The *limma* eBayes-moderated *t-*test was carried out using *M* values. No differences were observed between patients and controls regarding age and gender; therefore, no covariates were included in the model. DMRs with at least five CpGs and an FDR < 0.05 were considered statistically significant.

### Gene ontology (GO) and transcription factor (TF) enrichment analysis

Gene ontology (GO) evaluation was performed using the GREAT online tool (http://great.stanford.edu/public/html) [57], which accepts genomic regions as input, in our case DMRs, with the *two nearest genes* settings. Motif enrichment was analysed using the findMotifsGenome.pt tool of the HOMER motif discovery application [58], considering a window of ± 50 bp from each DMR. The total annotated DMRs from the mCSEA analysis were used as a background in both analyses. We considered significant enrichment for both GO and TF motif enrichment when *p* value < 0.05.

### Human Protein Atlas (HPA) gene expression and expression cluster data

We used public data from the HPA to evaluate the potential cerebral regional activity of enriched TF associated with cfDNA DMRs. To evaluate the expression levels of the genes associated with those factors, we used the *RNA consensus tissue gene data*, which consists of transcript expression levels summarized per gene in 55 tissues obtained consensually from RNA-seq data from the HPA and Genotype-Tissue Expression (GTEx) projects.

We also accessed data from the HPA consisting of the levels of confidence of protein-coding genes across tissue clusters and across single-cell clusters. For the tissue clusters, the HPA used RNA expression data from 53 tissues to classify genes into 87 expression clusters. A total of 144 cell types or cell lines were used to construct 68 single-cell expression clusters. For both approaches, Louvain clustering was performed based on gene-to-gene distances calculated from the Spearman correlation of gene expression across multiple samples. Clustering was performed 100 times to accommodate stochasticity. The confidence of the gene-to-cluster value, which varies between 0 and 1, corresponds to the proportion of times that a gene was assigned to a cluster. Clusters were identified manually through resource-to-functional annotation tools.

### Overlap of cfDNA DMRs with epileptic, neuron-specific and glia-specific DMRs

We extracted the lists of DMRs significantly altered in hippocampal and neocortical tissue in MTLE patients relative to autopsied non-epileptic controls, obtained in a previous study by our group [30]. We extracted raw IDAT files from 33 NeuN + fractions of prefrontal cortex of healthy individuals (GSE112179), 23 NeuN fractions (glia) (GSE166207) and 37 blood-cell-type samples, which included B cells, CD8+ T cells, CD4+ T cells, monocytes, neutrophils and NK cells (GSE110555). Data were processed and DMRs calculated as described above, all NeuN or glia samples being compared with all blood samples, without discrimination for blood cell types, and without considering any covariates. Since the *mCSEA* package was used to calculate DMRs across all settings, we overlapped the lists of DMRs based on their annotated identification. The *GeneOverlap* function, which is based on Fisher’s exact test, was used to calculate the significance of the overlap. The total number of DMRs considered by mCSEA was used as background (26,208 for promoter DMRs; 23,772 for gene DMRs; 27,187 for CGI-DMRs).

### DNase-seq data analysis

DNase hypersensitivity bigwig files with the human GRCh37 assembly from brain tissue and blood cells were obtained from ENCODE (Additional file 7: Table S4). For each setting, the mean function of Wiggletools was used to aggregate the multiple files [59]. The outputted wig file was reconverted to bigwig format using ucsc-wigtobigwig [60].

### Heatmaps, DMR visualization and plots

All heatmaps were developed using the R *gplots* and *ComplexHeatmap* [61] packages. Row dendrogram clustering was carried out with complete-linkage hierarchical clustering. The overlaps between lists of DMRs were represented using the *UpSet* function of *ComplexHeatmap*. To visualize individual DMRs, along with genomic location and epigenetic and chromatin accessibility marks, we used the functions available in the *gviz* package [62]. All additional plots were generated with the *ggplot2* package [63].

### Statistical analysis

All statistical analyses were done using R v4.0.2. or IBM SPSS Statistics version 27 (Armonk, NY, USA). All graphs were created in R. Group medians were compared using the Mann–Whitney test for numeric variables (age, percentage of cell/tissue type contribution). Fisher’s exact test was used to calculate the significance of non-random association between two categorical variables (sex distribution in patients and controls). The levels of significance were: *p < 0.05; **p < 0.01; ***p < 0.001.

## Supplementary Information

**Additional file 1.** Individual estimated percentages of contribution of each cell and tissue type from the meth_atlas deconvolution tool for the serum samples analysed (11 CTR and 12 MTLE–HS). **A** Distribution of all percentages of contributions of individual samples. **B** Boxplot comparison of the individual estimated proportion of cfDNA for the tissue and cell types within each study group. Vascular endothelial cell percentage contribution was significantly lower in MTLE–HS patients (n=12) than in CTR (n=11) (Wilcoxon test).*p < 0.05; **p < 0.01; ***p < 0.001.

**Additional file 2.** **A** Heatmap representation of gene-expression values (nTPM) of the protein-coding genes associated with enriched TF motifs in cfDNA DMRs across CNS tissues, extracted from the HPA. **B** Heatmap representation of the level of confidence of the protein-coding genes associated with enriched TF motifs in cfDNA DMRs across the brain-related tissue clusters, extracted from the HPA. **C** Heatmap representation of the level of confidence of the protein-coding genes associated with enriched TF motifs in cfDNA DMRs across the brain-related single-cell clusters, extracted from the HPA.

**Additional file 3** **A** Heatmap representation of DNA methylation of promoter, gene and CGI-DMRs in hippocampus of MTLE–HS patients compared with the hippocampus of controls. The DNA methylation value of each DMR corresponds to the mean beta value across the single-nucleotide positions encompassed by the DMR. Each individual is annotated with respect to age and sex. **B** Heatmap representation of DNA methylation of promoter, gene and CGI-DMRs in neocortex of MTLE–HS patients compared with the neocortex of controls. **C** Heatmap representation of DNA methylation of promoter, gene and CGI-DMRs in NeuN+ fractions from the prefrontal cortex of healthy individuals (GSE112179) compared with blood cells (GSE110555) **D** Heatmap representation of DNA methylation of promoter, gene, and CGI-DMRs in glial fractions from white matter of healthy individuals (GSE166207) compared with blood cells (GSE110555). **E** Representation of the overlap between cfDNA DMRs in MTLE–HS and neuron-specific DMRs. The sets of DMRs overlapped separately in relation to the type of DMR (promoter, gene, CGI) and to methylation behaviour (hypermethylated and hypomethylated). Red bars and lines correspond to the overlap of DMRs with coincidence in the direction of change. **F**. Heatmap matrix representation of the p values associated with the Fisher’s exact test of the overlaps between DMRs in cfDNA and neuron-specific DMRs. **G** Representation of the overlap between cfDNA DMRs in MTLE–HS glia-specific DMRs. The sets of DMRs overlapped separately in relation to the type of DMR (promoter, gene, CGI) and the methylation behaviour (hypermethylated and hypomethylated). Red bars and lines correspond to the overlap of DMRs with coincidence in the direction of change. **H** Heatmap matrix representation of the p values associated with the Fisher’s exact test of the overlaps between DMRs in cfDNA and glia-specific DMRs.

**Additional file. 4.** Meth_atlas output. Estimated cell and tissue proportions of contribution for all samples.

**Additional file. 5. **Meth_atlas output. Estimated cell and tissue proportions of contribution for all samples.

**Additional file 6.** Differentially methylated regions (DMRs) in cfDNA of MTLE patients in comparison with controls.

**Additional file 7.** List of DNase-seq data sets obtained from the ENCODE.

## Abbreviations

AD: Alzheimer’s disease
BDNF: Brain-derived neurotrophic factor
CAD: Caspase-activated DNase
cfDNA: Cell-free DNA
CGIs: CpG islands
CTR: Controls
DMR: Differentially methylated region
EEG: Electroencephalogram
GABA: γ-Aminobutyric acid
GO: Gene ontology
GTEx: Genotype tissue expression
HPA: Human Protein Atlas
HS: Hippocampal sclerosis
mCSEA: Methylated CpG set enrichment analysis
MS: Multiple sclerosis
MTLE: Mesial temporal lobe epilepsy
NET: Neutrophil extracellular traps
OPCs: Oligodendrocyte precursor cells
TBI: Traumatic brain injury
TF: Transcription factor

## Declarations

### Availability of data and materials

EPIC DNA methylation array data have been deposited in the NCBI’s Gene Expression Omnibus database under accession code GSE208758. All additional data used in this study are available in this article or in the supplementary information.
